# Performance of an In-House Human Immunodeficiency Virus Type 1 Genotyping System for Assessment of Drug Resistance in Cuba

**DOI:** 10.1371/journal.pone.0117176

**Published:** 2015-02-11

**Authors:** Yoan Alemán, Lore Vinken, Vivian Kourí, Lissette Pérez, Alina Álvarez, Yeissel Abrahantes, Carlos Fonseca, Jorge Pérez, Consuelo Correa, Yudira Soto, Yoeri Schrooten, Anne-Mieke Vandamme, Kristel Van Laethem

**Affiliations:** 1 Virology Department, Institute of Tropical Medicine “Pedro Kourí”, Havana City, Cuba; 2 Rega Institute for Medical Research, Department of Microbiology and Immunology, KU Leuven, Leuven, Belgium; 3 Hospital at Institute of Tropical Medicine “Pedro Kourí”, Havana City, Cuba; 4 Centro de Malária e outras Doenças Tropicais and Unidade de Microbiologia, Instituto de Higiene e Medicina Tropical, Universida de Nova de Lisboa, Lisboa, Portugal; Meharry Medical College, UNITED STATES

## Abstract

As commercial human immunodeficiency virus type 1 drug resistance assays are expensive, they are not commonly used in resource-limited settings. Hence, a more affordable in-house procedure was set up taking into account the specific epidemiological and economic circumstances of Cuba. The performance characteristics of the in-house assay were evaluated using clinical samples with various subtypes and resistance patterns. The lower limit of amplification was determined on dilutions series of 20 clinical isolates and ranged from 84 to 529 RNA copies/mL. For the assessment of trueness, 14 clinical samples were analyzed and the ViroSeq HIV-1 Genotyping System v2.0 was used as the reference standard. The mean nucleotide sequence identity between the two assays was 98.7% ± 1.0. Additionally, 99.0% of the amino acids at drug resistance positions were identical. The sensitivity and specificity in detecting drug resistance mutations was respectively 94.1% and 99.5%. Only few discordances in drug resistance interpretation patterns were observed. The repeatability and reproducibility were evaluated using 10 clinical samples with 3 replicates per sample. The in-house test was very precise as nucleotide sequence identity among paired nucleotide sequences ranged from 98.7% to 99.9%. The acceptance criteria were met by the in-house test for all performance characteristics, demonstrating a high degree of accuracy. Subsequently, the applicability in routine clinical practice was evaluated on 380 plasma samples. The amplification success rate was 91% and good quality consensus sequences encoding the entire protease and the first 335 codons in reverse transcriptase could be obtained for 99% of the successful amplicons. The reagent cost per sample using the in-house procedure was around € 80 per genotyping attempt. Overall, the in-house assay provided good results, was feasible with equipment and reagents available in Cuba and was half as expensive as commercial assays.

## Introduction

Access to antiretroviral therapy (ART) has been scaled up rapidly since 2001 in Cuba and this has markedly reduced human immunodeficiency virus (HIV)/Acquired Immune Deficiency Syndrome (AIDS) related morbidity and mortality [1]. By the end of 2013, 58.6% of people living with HIV-1 were receiving ART (unpublished data). Due to financial constraints, viral load testing was only implemented in 2003 and frequency of CD4 and viral load testing was lower than recommended in international guidelines [2]. This creates the potential of on-going viral replication resulting into emergence and transmission of drug resistance which necessitates drug resistance testing for surveillance purposes and for individual patient monitoring [3–5]. Therefore, financial support for the implementation of HIV-1 drug resistance testing was requested to the Vlaamse Interuniversitaire Raad (VLIR) and granted in 2008.

The aim of this study was to design an accurate and affordable HIV-1 genotyping system enabling the assessment of drug resistance against protease (PR) and reverse transcriptase (RT) inhibitors in the Cuban setting. The Cuban HIV-1 epidemic is characterized with the presence of almost all subtypes and particular circulating recombinant forms (CRFs). In 2003, the highest prevailing subtype was subtype B (41%), followed by CRF19_cpx (18%), BG recombinants (12%), CRF18_cpx (7%), subtype C (6%), subtype G (4%), subtype H (2%) and other subtypes and unique recombinant forms (URFs) (10%)[6]. The performance characteristics of the drug resistance assay and its applicability were also evaluated.

## Material and Methods

### Clinical samples

A total of 424 clinical plasma samples was used in the validation of the in-house assay.

A selection of 20 plasma samples from HIV-1 patients attending the Institute of Tropical Medicine “Pedro Kourí” (IPK) (Cuba) was used to determine the lower limit of amplification. The panel included the predominant subtypes in Cuba and comprised subtypes A1 (2 samples), B (4), C (4), CRF18_cpx (4), CRF19_cpx (4), and BG-recombinants (2). Viral loads were determined using Nuclisens Easy Q HIV-1 Kit version 2.0 (Biomérieux, Craponne, France) or COBAS TaqMan HIV-1 Test For Use With The High Pure System (Roche, Mannheim, Germany).

To assess the trueness (including sensitivity and specificity), 14 plasma samples from HIV-1 patients attending the University Hospitals in Leuven (Belgium) were selected based upon sequences previously obtained at the University Hospitals of Leuven (Belgium) using the ViroSeq HIV-1 genotyping system v2.0 (Celera, Alameda, CA, USA; Abbott Molecular, Wavre, Belgium). The panel included samples with subtypes A (2 samples), B (4), C (1), D (1), G (1), H (1), CRF02_AG (1) and URF (3), representing multiple resistance patterns including mixtures and viral loads ranging from 692 to 263,027 RNA copies/mL. Viral loads were determined using the Abbott RealTime HIV-1 assay (Abbott Molecular, Wavre, Belgium).

To evaluate the precision of the assay, repeatability and reproducibility were determined on 2 panels of 5 plasma samples from HIV-1 patients attending the IPK and displaying multiple drug resistance patterns and various subtypes (subtypes B (2), C (2), CRF18_cpx (2), CRF19_cpx (1), CRF20_BG (1), CRF24_BG (2)). The selection of patients was based upon sequences previously obtained at the IPK using the in-house drug resistance test described in this study.

For the evaluation of the applicability of the test in a clinical setting, the genotyping results were evaluated for 380 plasma samples from 352 HIV-1 patients attending the IPK for clinical care between September 2009 and September 2011. This dataset included 92 plasma samples obtained from therapy naïve patients and 220 samples from therapy experienced patients. For the remaining 68 plasma samples, the therapy history of the patients was unknown.

### In-house genotyping system

1. RNA extraction

For HIV-1 viral RNA extraction, 1 mL of plasma or serial dilutions of plasma in phosphate buffered saline (PBS) was ultra-centrifuged at 20,000 x g for 1 h at 4⁰C to pellet the virus. The supernatant was removed, retaining 140 μL to suspend the pellet again. Subsequently, the viral RNA extraction was performed manually or automatically on the QIAcube (QIAGEN, Hilden, Germany) using the QIAamp Viral RNA Mini Kit (QIAGEN, Hilden, Germany), as described by the manufacturer’s protocol. Isolated RNA was eluted in 60 μL elution buffer and stored at -80°C until use.

2. Primer design

The PCR and sequencing primers were designed using the *gag-pol* gene alignment of several HIV-1 group M strains available at the Los Alamos HIV Database (www.hiv.lanl.gov), with the inclusion of all available strains from CRFs in Cuba (CRF18_cpx, CRF19_cpx, CRF20_BG, CRF23_BG and CRF24_BG) [6]. The primers were positioned taking into account the HIV-1 genetic variability, the absence of codons associated with drug resistance and the average coverage length of sequencing segments obtained with the local automatic sequencer (CEQ 8800 Genetic Analysis System (Analis, Namen, Belgium)). The primers were evaluated using OLIGO software (Medprobe, Oslo, Norway) and modified when necessary. The primers were synthesized by Life Technologies (Gent, Belgium). The primer sequences are given in Table 1.

**Table 1:** Primers for the amplification and sequencing of the HIV-1 protease and reverse transcriptase.

Primers	Sequence 5’-3’	Position ^a^	Direction	Description
AV159	GGG GTT AAA TAA AAT AGT AAG	1593–1613	Sense	Outer primer for amplification
AV192	AAT TGT TTT ACA TCA TTA GTG TG	3630–3652	Antisense	Outer primer for amplification
AV190	GCT ACA CTA GAA GAA ATG ATG AC	1810–1832	Sense	Inner primer for amplification
AV191	CTT GAT AAA TTT GAT ATG TCC ATT G	3555–3579	Antisense	Inner primer for amplification
KVL162	TTC CCT CAR ATC ACT CTT TGG CA	2250–2272	Sense	Sequencing
KVL164	ACC AGT AAA AYT RAA RCC AGG AAT G	2573–2597	Sense	Sequencing
KVL163	CAC AGG GAT GGA AAG GRT CAC C	2998–3019	Sense	Sequencing
KVL177	AAR GAM AGC TGG ACT GTC AAT GA	3294–3316	Sense	Sequencing
KVL165	GTR TTR TAT GGA TTT TCA GGC CCA A	2698–2722	Antisense	Sequencing
KVL176	TTG YTC TAT GYT GCC CTA TTT CTA	3127–3150	Antisense	Sequencing
KVL178	CTA TTA AGT CTT TTG ATG GGT CAT A	3504–3528	Antisense	Sequencing

^a^ Positions according to HXB2 (GenBank accession number K03455).

3. Amplification

Ten microliters of the extracted RNA was reverse transcribed and amplified in a one-step RT-PCR using the SuperScript III One-Step RT-PCR System with Platinum Taq High Fidelity kit (Life Technologies, Gent, Belgium). The reaction mixture was composed by: 25 μL of 2X Reaction Mix (containing 0.4 mM of each dNTP and 2.4 mM MgSO_4_), 0.5 μL 20 μM primer AV159 (Table 1), 0.5 μL 20 μM primer AV192 (Table 1), 8 μL 5 mM MgSO_4_, 1 μL SuperScript III RT/Platinum Taq High Fidelity Enzyme Mix, 0.25 μL 40 U/μL Protector RNase inhibitor (Roche, Mannheim, Germany) and H_2_O until a final volume of 40 μL. Reverse transcription was performed at 55°C for 30 min. The amplification started with an initial denaturation at 94°C for 2 min, followed by 40 cycles of: denaturation at 94°C for 15 sec, annealing at 50°C for 30 sec and elongation at 68°C for 2 min, with a final elongation at 68°C for 5 min. A 2,060-bp amplicon, encompassing HIV-1 PR and the first part of RT, was thus obtained.

The inner 1,770-bp PCR fragment was generated using the Expand High Fidelity PCR System (Roche, Mannheim, Germany). To prevent degradation of primers and template, the PCR mixes were prepared in two separate tubes. Thus, a first tube was prepared containing 0.4 μL 25 mM of each dNTP, 1.25 μL 20 μM primer AV190 (Table 1), 1.25 μL 20 μM primer AV191 (Table 1) and H_2_O until a final volume of 23 μL. The second tube contained 5 μL 10X Expand High Fidelity Buffer, 6 μL 25 mM MgCl_2_, 0.75 μL 3.5 U/μL Expand High Fidelity Enzyme Mix and H_2_O until a final volume of 25 μL. Two microliter of the outer PCR product was added to the first tube and subsequently 25 μL of the second PCR mix was added to each reaction tube, immediately before starting the cycling program. This reaction was subjected to 95°C for 2 min; 30 cycles of 95°C for 15 sec, 50°C for 30 sec and 68°C for 2 min; and finally to 68°C for 5 min. All cycling programs were run on a Thermocycler Doppio (VWR, Leuven, Belgium). Amplification products were separated on a 1% agarose gel and visualized by ethidium bromide staining and UV light. Only positive reactions were subjected to sequencing analysis.

4. Sequencing analysis

The PCR products were purified using the QIAquick PCR Purification Kit (QIAGEN, Hilden, Germany) following the protocol indicated by the manufacturers. Purified PCR products were subjected to population-based bi-directional sequencing using the Dye Terminator Cycle Sequencing (DTCS) Quick Start Kit (Analis, Namen, Belgium). Separate reactions with 7 different primers were performed, in order to enable the coverage of a 1,302-bp fragment that encodes the 99 amino acids of HIV-1 PR and the first 335 amino acids of HIV-1 RT. Each mixture consisted of 1 μL 5 μM primer (KVL162, KVL163, KVL164, KVL165, KVL176, KVL177 or KVL178) (Table 1), 8 μL Quick Start DTCS Master Mix, 5 μL purified PCR product and 6 μL H_2_O to complete the reaction to 20 μL. Cycling conditions on a Thermocycler Doppio were 50 cycles of 96°C for 30 sec, 50°C for 20 sec and 60°C for 4 min. The sequence products were purified according to the protocol described in the DTCS Quick Start Kit. The sequencing fragments were run on a CEQ 8800 Genetic Analysis System (Analis, Namen, Belgium). Finally, the electropherograms were displayed and assembled, and sequences were manually edited using Sequencher version 4.10.1 (Gene Codes Corporation, Ann Arbor, USA) and HIV-1 subtype B strain HXB2 as a reference (GenBank accession number K03455).

### ViroSeq HIV-1 Genotyping System

The FDA-cleared and CE-marked ViroSeq HIV-1 Genotyping System version 2.0 (Celera, Alameda, CA, USA; Abbott Molecular, Wavre, Belgium) was used as the standard test for the validation of the in-house assay. ViroSeq is designed to detect drug resistance mutations (DRM) in the entire PR (amino acids 1 to 99) and the first part of the RT gene fragment (amino acids 1 to 335) from HIV-1 subtype B plasma samples with a viral load ranging from 2,000 to 750,000 RNA copies/mL (ViroSeq specifications). At a viral load of 2,000 RNA copies/mL and with 40% mutant viruses present, the sensitivity and specificity in detecting HIV-1 DRM was 98.1% and 99.9%, respectively. These values decreased to respectively 92.7% and 99.9% for samples with a viral load of 1,000 RNA copies/mL and 40% mutant viruses (ViroSeq specifications). For clinical samples with a viral load ranging from 1,800 to 10,500 RNA copies/mL, the sensitivity and specificity at a threshold of 40% mutants was 99.65% and 99.95%, respectively [7]. The repeatability and reproducibility for detecting DRM were ≥95% (ViroSeq specifications).

In this study, ViroSeq testing was performed at the AIDS Reference Laboratory in Leuven (Belgium), where this system has been used since 2000 for monitoring drug resistance in HIV-1 infected patients [8]. All steps in the procedure were performed according to manufacturer’s instructions, except for plasma volume, non-B subtypes and sequencing editing software. Indeed, RNA extraction was performed using 1 mL of plasma to increase the amplification sensitivity and to enable genotyping below 2,000 RNA copies/mL [9]. The sequencing reactions were run on an ABI3130xl Genetic Analyzer (Life Technologies, Gent, Belgium), and electropherograms were assembled and subsequently manually edited using SeqScape version 2.6 (Life Technologies, Gent, Belgium). The AIDS Reference Laboratory yearly participates in an external quality assessment program (ENVA HIV Drug Resistance Typing panels from QCMD (Quality Control for Molecular Diagnostics, Glasgow, UK)) for which excellent results have been obtained for trueness [10]. Nucleotide (NT) and amino acid (AA) identity scores at DRM positions were respectively 99.76% and 99.67% when compared to the reference sequence, which was the consensus sequence derived from the results of all submitting participants within the program.

### Performance evaluation

In order to assess the performance of the in-house genotyping assay, the lower limit of amplification and the accuracy (including trueness and precision) in detecting NT and AA sequences as well as in detecting mutations at DRM positions were evaluated. The general definitions of these performance characteristics were adopted from Jennings et al. [11]. Acceptance criteria for some of the characteristics were established prior to analysis (see further).

The lower limit of amplification was defined as the lowest viral load at which samples could be amplified. Briefly, plasma samples were 10-fold serially diluted in PBS until a theoretical viral load below 1,000 RNA copies/mL was achieved. The serial dilutions were extracted and amplified with the in-house test. The procedure was considered successful if the amplicons could be visualized with ethidium bromide staining and UV light on an agarose gel. Subsequently, the last serial dilution that was considered positive, was subjected to 2-fold serial dilutions until a theoretical viral load below 100 RNA copies/mL was achieved, extracted, amplified and analyzed on an agarose gel. The lower limit of amplification should be ≤1,000 RNA copies/mL as this is the cut-off for which drug resistance testing is recommended [2].

Trueness was assessed using 14 clinical samples and one negative control, and by comparing genotyping results of the in-house assay with the results of the ViroSeq HIV-1 Genotyping System version 2.0. The acceptance criterion for trueness was established as the pairwise agreement of ≥95% at NT level. At AA level, the threshold was defined as ≥99% identity at known DRM positions if no mixtures are present and >50% if mixtures are present in the reference standard [5,12–14].

Precision (including repeatability and reproducibility) was assessed using 10 clinical samples with 3 replicates per sample. More specifically, the repeatability was determined using 5 samples for which the 3 replicates were generated by a single technician in a single run. The reproducibility was determined using 5 samples, of which one sample was diluted in PBS until theoretical viral loads of 2,000; 1,000; 500 and 250 RNA copies/mL. The latter experiment was performed to assess the reproducibility at low viral loads. For each aliquot, 3 replicates were generated in 3 separate runs and by 2 technicians. The acceptance criterion for repeatability and reproducibility was defined as ≥98% NT identities in ≥90% of the pairwise comparisons [9,12,14,15].

Nucleotide sequence identity scores were defined as the percentage NT identity of a pairwise comparison of NT sequences. Mixed versus unmixed bases were counted as discordant. The NT sequence identity scores were determined using the EMBOSS pairwise alignment tool (http://www.ebi.ac.uk/Tools/psa/emboss_needle/nucleotide.html) with default gap-penalty values. Amino acid sequence identity scores were defined as the percentage AA identity of a pairwise comparison of AA sequences. AA identity scores were also determined at DRM positions and at surveillance drug resistance mutation (SDRM) positions only. Calculations were performed using SeqScape version 2.6. Mixed versus unmixed AA were counted as discordant. DRM positions were defined as all positions for which AA changes are included in the resistance interpretation algorithms Rega v9.1.0, ANRS 2013.09 and/or HIVDB v7.0 (89 DRM positions). SDRM positions were defined according to Bennett et al. [16] (43 SRDM positions).

Drug resistance interpretations were conducted using the Stanford website and resistance interpretation algorithms Rega v9.1.0, ANRS 2013.09 and/or HIVDB v7.0 (http://sierra2.stanford.edu/sierra/servlet/JSierra?action=algSequenceInput).

### Subtyping

HIV-1 subtyping was performed using the Rega subtyping tool version 3 [17], and confirmed by manual phylogenetic analysis using CLUSTAL-X and the neighbor-joining method in MEGA version 5 (Kimura’s 2-parameter correction, bootstrap 1,000) [18]. The assignment of recombinant genetic forms was done using Simplot version 2.5 [19]. The reference dataset included subtypes A (Genbank accession number AM000055, AM000053), B (AY037269, DQ383746, K03455), C (AY563170, AY727522), D (K03454, M27323), F (DQ189088, AF077336), G (AF084936, AY371121), H (AF190127, AF190128, AF005496), J (EF614151, AF082394, AF082395), K (AJ249235, AJ249239), recombinants containing unclassified regions (AF286236, AF457101), CRF01_AE (AF197340, U51188), CRF02_AG (AY371122, AY371123), CRF18_cpx (AF377959, AY586541, AY894993, AY586540), CRF19_cpx (AY588971, AY588970, AY894994), CRF20_BG (AY586545, AY586544), CRF23_BG (AY900571, AY900572) and CRF24_BG (AY900574, AY900575).

### Quality control

A negative control was included in each run to monitor for cross-contamination. Additionally, to confirm the absence of any sample mix-up and/or cross-contamination, sequence artifacts were investigated using the Calibrated Population Resistance tool version 6.0 (http://cpr.stanford.edu/cpr.cgi) and phylogenetic reconstructions were performed on all generated sequences. The 2008 subtypes and CRF reference dataset from Los Alamos database (http://www.hiv.lanl.gov/content/sequence/NEWALIGN/align.html) was included. The sequences were aligned with Muscle in MEGA version 5. Surveillance drug resistance mutation (SDRM) positions [16] were removed from the alignment to avoid bias from convergent evolution [20]. A maximum likelihood tree was constructed using the RAxML v7.8.1 software [21] with the GTR+г NT substitution model. The robustness of the tree topology was assessed by a bootstrap analysis of 1,000 replicates. The tree diagram was plotted using FigTree v1.4.0.

### Ethics statement

The study has been approved by the ethical committee of the Institute of Tropical Medicine “Pedro Kourí” (Havana, Cuba), and complies with the principles laid down in the Declaration of Helsinki. All subjects included in the study, gave written informed consent to the work.

## Results

### Lower limit of amplification

The lower limit of amplification ranged between 84 and 529 RNA copies/mL, and the median limit was 275 RNA copies/mL (IQR 225–363) (Table 2). For all subtypes the lower limit of amplification was well below the recommended threshold of 1,000 RNA copies/mL.

**Table 2:** Lower limit of amplification of the in-house HIV-1 genotyping assay.

Subtype	Sample	HIV-1 RNA copies/mL	
		Original viral load	Lower limit of amplification
A1	1	33,333	84
	2	70,000	350
B	3	52,873	529
	4	12,000	300
	5	3,009,000	301
	6	1,000,000	500
C	7	15,000	375
	8	15,000	375
	9	50,574	253
	10	200,000	200
CRF18_cpx	11	11,000	138
	12	16,000	160
	13	465,507	466
	14	80,000	100
CRF19_cpx	15	1,100,000	275
	16	11,000	275
	17	2,000	250
	18	499,989	250
BG-recombinants	19	114,940	287
	20	26,000	260

### Trueness

Trueness was assessed in each specimen of a set of 15 samples, by comparing genotyping results obtained with the reference method ViroSeq and the in-house assay (Table 3). One negative control was included in the panel and was negative in the in-house test. The viral loads of the 14 clinical samples ranged from 692 to 236,027 RNA copies/mL. Genotyping was successful for all these samples with both assays. For specimen 3 and 14, the NT sequences generated by ViroSeq were shorter than anticipated (respectively 1,218 and 1,221 NT), so only 88 of 89 predefined DRM positions could be evaluated for these samples.

**Table 3:** Assessment of trueness in each specimen for the in-house HIV-1 genotyping system as compared to the ViroSeq system.

Specimen ID	Subtype	Viral load (copies/mL)	Mutations at (S)DR pos.^a^			Sequence identity score (%)		AA identity score (%)	
			Assay	PR	RT	NT	AA	At DR pos.	At SDR pos.
01	A/G	2,455	VS	20I, 35D, 36I, 41K, 69K, 70KR, 77V, **82I**, 89M	-	98.0	97.9	97.8	100.0
			IH	20I, 35D, 36I, 41K, 69K, 70K, 77IV, **82I**, 89M	-				
02	H	28,184	VS	16E, 20R, 36I, 41K, 43KR, 60E, 62V, 63N, 69K, 89I	-	96.7	97.2	98.9	100.0
			IH	16E, 20R, 36I, 41K, 43K, 60E, 62V, 63N, 69K, 89I	-				
03	D/B	2,884	VS	16A, 41N, 69Q, 77I	40D, 189I	98.9	99.1	100.0	100.0
			IH	16A, 41N, 69Q, 77I	40D, 189I				
04	B	5,129	VS	35D, 63P, 93L	-	98.7	99.3	100.0	100.0
			IH	35D, 63P, 93L	-				
05	D	158,489	VS	10V, 16E, 41K, 43K, 60E, 62V, 64IL, 77I, **85VI**, 89F, 93L	-	96.9	97.0	97.8	100.0
			IH	10V, 16E, 41K, 43EK, 60E, 62V, 64IL, 77I, **85VI**, 89F, 93IL	-				
06	B	1,660	VS	10IL, 35D, 36IM, 41KR, **54IV**, 62IV, 63T, 71AV, **84IV**, **90LM**	**74LV**, 98AS, **115FY**, **184MV**, 221HY	98.6	98.4	96.6	100.0
			IH	10I, 35D, 36IM, 41R, **54IV**, 62IV, 63T, 71AV, **84IV**, **90LM**	**74LV**, 98AS, **115FY**, **184MV**, 221H				
07	G	42,658	VS	20I, 35Q, 36I, 41K, 63P, 69K, **82I**, 89M	-	99.2	100.0	100.0	100.0
			IH	20I, 35Q, 36I, 41K, 63P, 69K, **82I**, 89M	-				
08 ^b^	-	0	VS	-	-	-	-	-	-
			IH	-	-				
09	B	8,318	VS	15V, 35D, 63P	-	99.9	99.8	100.0	100.0
			IH	15V, 35D, 63P	-				
10	C	10,965	VS	15V, 36I, 41N, 63LP, 69K, 89LM, 93L	40D	98.8	99.1	100.0	100.0
			IH	15V, 36I, 41N, 63LP, 69K, 89LM, 93L	40D				
11	B/D	236,027	VS	15V, 20R, 35D, 36I, 41KR, **47** **I**, **50** **I**, **54IV**, 62V, 63P, 69H, 71V, **88D**	**210W**, **215S**	98.6	97.9	96.6	95.3
			IH	15V, 20R, 35D, 36I, 41KR, **47** **I** **M**, **50** **I** **M**, **54IV**, 62V, 63P, 69HN, 71V, **88D**	**210W**, **215S**				
12	CRF02_AG	1,413	VS	20I, 36I, 41K, 69K, 70R, 89M	**67** **D** **N**, **106A**	99.4	98.8	98.9	97.7
			IH	20I, 36I, 41K, 69K, 70R, 89M	**67** **D**, **106A**				
13	A	692	VS	35D, 36I, 41K, 69K, 70R, 89M	40D, **103N**, **179I**	100.0	100.0	100.0	100.0
			IH	35D, 36I, 41K, 69K, 70R, 89M	40D, **103N**, **179I**				
14	A	18,621	VS	20KR, 33F, 36I, 41K, 69K, 89M, 93L	**67N**, **69N**, **179IV**, **219Q**	98.9	99.3	98.9	100.0
			IH	20R, 33F, 36I, 41K, 69K, 89M, 93L	**67N**, **69N**, **179IV**, **219Q**				
15	B	66,069	VS	20R, 36ILM, 45KR, 62IV, 63AP, 77I, 93L	41L	99.3	99.5	100.0	100.0
			IH	20R, 36ILM, 45KR, 62IV, 63AP, 77I, 93L	41L				

ID identity; AA amino acid; NT nucleotide; (S)DR pos. (surveillance) drug resistance positions; VS ViroSeq; IH in-house; PR protease; RT reverse transcriptase.

^a^ AA changes at DR pos. using HXB2 (GenBank accession number K03455) as a reference sequence. Bold: SDR pos., underlined: discordances between the ViroSeq and in-house assay.

^b^ Sample 08 was the blinded negative control.

The mean NT sequence identity score between both assays was 98.7% ± SD 1.0 (trueness). Each individual NT sequence identity score was well above the predefined acceptance criterion of ≥95%. Among the 14 samples, 235 NT differences were observed between both assays and were in 98% of the instances caused by a difference in the detection of mixtures (230/235). The other discordances (5/235) resulted from scoring a different nucleotide. In total, 486 NT mixtures were identified by the ViroSeq system and/or the in-house assay. From these mixtures, 256 (53%) were detected by both assays, whereas 150 (31%) were only detected by ViroSeq and 80 (16%) only by the in-house assay.

The overall AA identity score at DRM positions was 99.0% (trueness) as 1,231 of the 1,244 evaluated DRM positions were identical for both methods (Table 4). Furthermore, 99.5% agreement was observed at DRM positions if no mixtures were present (1,210 of 1,216 positions) and 75% concordance at DRM positions where mixtures were present (21 of 28 positions) in the ViroSeq result. At SDRM positions, 599 of the 602 evaluated positions were concordant between the two assays, resulting in an overall trueness of 99.5%. The agreement at SDRM positions was 99.7% if no mixtures were present (590 of 592 positions) and 90% if mixtures were present (9 of 10 positions) in the ViroSeq result. At both DRM and SDRM positions, the predefined acceptance criteria for trueness were met. The AA differences at DRM and SDRM positions were all caused by mixtures detected by one of the methods, and not by the other. The in-house method detected mixtures at 6 of the 13 discordant DRM positions and at 2 of the 3 discordant SDRM positions, whereas ViroSeq detected mixtures at 7 and 1 positions, respectively. In total, 33 AA mixtures at DRM and 12 AA mixtures at SDRM positions were identified by the ViroSeq system and/or the in-house assay. From these mixtures, 20 (61%) at DRM and 9 (75%) at SDRM positions were detected by both assays, whereas respectively 7 (21%) and 1 (8%) only by ViroSeq and 6 (18%) and 2 (17%) only by the in-house assay.

**Table 4:** Overall assessment of trueness in detecting mutations at (surveillance) drug resistance positions for the in-house HIV-1 genotyping system.

		No. of mutations at (S)DR pos. by ViroSeq						
				%				
	In-house	Positive	Negative	Sensitivity	Specificity	Trueness	PPV	NPV
DR pos.	Positive	111	6	94.1	99.5	99.0	94.9	99.4
	Negative	7	1,120					
SDR pos.	Positive	21	2	95.5	99.7	99.5	91.3	99.8
	Negative	1	578					

ViroSeq was used as the reference standard. (S)DR pos. (surveillance) drug resistance positions; PPV positive predictive value; NPV negative predictive value.

The specificity and sensitivity of the in-house assay were respectively 99.5% and 94.1% in detecting mutations at DRM positions and 99.7% and 95.5% in detecting mutations at SDRM positions (Table 4). In addition, the in-house assay demonstrated good positive and negative predictive values, respectively 94.9% and 99.4% at DRM positions and 91.3% and 99.8% at SDRM positions.

Only 3 DRM (43R in PR; 67N and 221Y in RT) observed in the ViroSeq results were not detected by the in-house assay. The other differences observed at DRM positions were related to amino acid changes that are not included in the drug resistance interpretation rules. By not detecting 221Y in RT, drug resistance was missed for rilpivirine according to ANRS and Rega (from respectively resistant and intermediate resistant in the ViroSeq result towards susceptible in the in-house result). By not detecting 67N in RT, intermediate resistance was not detected for zidovudine and stavudine according to HIVDB. The differences for PR mutation 43R did not alter the drug resistance interpretations. In addition, 2 DRM (69N and 77I in PR) were detected by the in-house method, but not by ViroSeq. Only PR mutation 69N altered the ANRS drug resistance interpretation for tipranavir/r (intermediate resistance in the in-house result and susceptible in the ViroSeq result). Finally, one SDRM (67N in RT) was detected by ViroSeq, but missed by the in-house method.

### Precision

The in-house assay showed good results for both repeatability (Table 5) and reproducibility (Table 6). In the assessment of reproducibility for sample 21, only 2 replicates could be generated. For sample 25, the amplification failed three times at the dilution step of 250 copies/mL.

**Table 5:** Repeatability of the in-house HIV-1 genotyping system.

Specimen ID	Subtype	Mutations at (S)DR pos.^a^		Mean sequence identity score (% ± SD)		Mean AA identity score (% ± SD)	
		PR	RT	NT	AA	At DR pos.	At SDR pos.
16	CRF18_cpx	16Q, 20I, 35G, 36I, 41K, **46I**, 69K, 70R, 71T, 77IV, **82I**, 89V, **90M**	**41L**, **69D**, **103N**, **181F**, **184V**, **210W**, **215Y**, **219R**, 221Y	99.9 ± 0.1	99.9 ± 0.1	100.0 ± 0.0	100.0 ± 0.0
17	C	10F, 20I, 35EG, 36I, 41K, **46I**, 62IV, 63P, 64IV, 69K, 71AITV, 74PT, 89M, **90M**, 93L	**67N**, **70R**, 98G, **101E**, **184V**, **190A**, **215F**, **219Q**	99.9 ± 0.1	99.9 ± 0.1	99.3 ± 0.6	100.0 ± 0.0
18	B	20R, 36I, 41K, 45R, 62V, 63P, 69Q	-	99.9 ± 0.1	100.0 ± 0.0	100.0 ± 0.0	100.0 ± 0.0
19	CRF20_BG	20I, 36I, 41K, 63S, 69K, 70KR, **82I**, 89M	40*E, **41L**, **67N**, **70R**, **106A**, **184V**, **210W**, **215F**, **219E**, 227L	99.8 ± 0.1	99.9 ± 0.1	100.0 ± 0.0	100.0 ± 0.0
20	CRF19_cpx	**30N**, 35DE, 36I, 41K, 43R, 45KQ, 63HP, 64IV, 69Q, 71AV, **88D**, 93IL	**74L** **V**, **103N**, **184V**, **215Y**, 318F	98.8 ± 0.2	98.2 ± 0.5	95.5 ± 1.1	98.5 ± 1.3

Number of replicates for each sample is 3. ID identity; AA amino acid; NT nucleotide; (S)DR(pos.) (surveillance) drug resistance (positions); SD standard deviation; PR protease; RT reverse transcriptase.

^a^AA changes at DR pos. using HXB2 (GenBank accession number K03455) as a reference sequence. Bold: SDR pos., underlined: discordant between the 3 replicates.

**Table 6:** Reproducibility of the in-house HIV-1 genotyping system.

Specimen ID	Subtype	Mutations at (S)DR pos.^a^		Mean sequence identity score (% ± SD)		Mean AA identity score (% ± SD)	
		PR	RT	NT	AA	At DR pos.	At SDR pos.
21	C	15V, 35D, 36I, 41K, 69K, **88NT**, 89M	**41L**, **75I**, 98S, **103N**, 132L, **184V**, **215Y**	99.6	99.5	100.0	100.0
22	CRF24_BG	10I, 15V, 20I, 35D, 36I, 41K, **54V**, 62V, 66F, 69K, 70R, 74K, **82I**, 89M, 93IV	62V, **70E**, **75I**, 98S, **115F**, **181C**, **184V**	99.7 ± 0.1	99.5 ± 0.3	100.0 ± 0.0	100.0 ± 0.0
23	CRF18_cpx	16E, 20I, 35D, 36I, 41K, 64M, 69K, 70R, 89M	40D, **181C**, **184V**, 189IV, 221Y	99.6 ± 0.1	99.7 ± 0.2	99.3 ± 0.6	100.0 ± 0.0
24	B	10I, 20IT, 33I, 35D, 36I, 41KR, 43T, **54V**, 60EQ, 62V, 63P, 64IL, 66F, 71V, **73A**, **82AT**, **84V**, 89V, **90M**	**41L**, **67N**, **69D**, **70R**, **74I**, 118I, **179I**, **184V**, **188L**, **190A**, **215F**, **219Q**	99.7 ± 0.1	99.9 ± 0.1	99.3 ± 0.6	100.0 ± 0.0
25–2000	CRF24_BG	20I, 35D, 36IV, 38IL, 41K, 69K, **82I**, 89M	98S	98.8 ± 0.4	99.4 ± 0.4	98.5 ± 1.3	100.0 ± 0
25–1000	CRF24_BG	20I, 35D, 36I, 41K, 69K, 70KR, **82I**, 89M	68NS, 98S	99.2 ± 0.2	99.1 ± 0.2	98.5 ± 1.3	100.0 ± 0
25–500	CRF24_BG	20I, 35D, 36I, 41K, 69K, **82I**, 89M	98S	98.7 ± 0.3	99.6 ± 0.2	100.0 ± 0	100.0 ± 0

Number of replicates for each sample is 3, except for specimen 21, for which only 2 replicates could be generated. Specimen identity (ID) 25–2000, 25–1000 and 25–500: serial dilutions of sample 25 until a theoretical viral load of respectively 2,000, 1,000 and 500 RNA copies/mL. AA amino acid; NT nucleotide; (S)DR(pos.) (surveillance) drug resistance (positions); SD standard deviation; PR protease; RT reverse transcriptase.

^a^AA changes at DR pos. using HXB2 (GenBank accession number K03455) as a reference sequence. Bold: SDR pos., underlined: discordant between the 3 (or 2) replicates.

The NT sequence identity ranged from 98.8% to 99.9% and from 98.7% to 99.7% in the repeatability and reproducibility assessment, respectively. For both analyses, 100% of the pairwise comparisons (15/15 and 20/20) were ≥98% identical on NT level, well above the predefined threshold of ≥98% nucleotide identities in ≥90% of the pairwise comparisons. At AA level, similarly high sequence identity scores were reached at all positions, DRM positions and SDRM positions, as shown in Tables 5 and 6. No differences were observed in the detection of SDRM between all the replicates of each sample for both repeatability and reproducibility.

Although in 2 samples not all mutations at DRM positions were detected in every replicate during the assessment of repeatability, this had only a minor impact on drug resistance interpretation, because the particular amino acid changes were not included in the drug resistance interpretation rules or they did not trigger a change in the interpretation. In total, only 2 DRM were not detected in the 3 replicates. In sample 17, the minor DRM 10F (in PR) was not detected in 2 replicates, resulting in an altered REGA and ANRS drug resistance interpretation for atazanavir/r (from resistant in the ViroSeq result towards respectively intermediate resistant and susceptible in the in-house result), an altered ANRS interpretation for fosamprenavir/r (from resistant towards susceptible) and a different ANRS resistance interpretation for saquinavir/r (from resistant towards intermediate resistant). In sample 20, the minor DRM 71V (in PR) was missed in 2 replicates, altering the REGA resistance interpretation for saquinavir/r (from intermediate resistant towards susceptible).

In the assessment of reproducibility, only two DRM were not detected in the 3 replicates. 189I in RT was missed in the replicate generated by the second technician for sample 23. For sample 25–2000, 36V in PR was detected by only one of the replicates. However, the absence of 189I and 36V did not change the drug resistance interpretations for these samples.

Finally, the sequences generated from 25–2000, 25–1000 and 25–500 were compared with the sequence generated from the original non-diluted plasma sample and the respective mean pairwise NT identity scores were 98.9% ± 0.6, 99.1% ± 0.2 and 99.0% ± 0.3. Mean pairwise AA identity scores were respectively 99.4% ± 0.5, 99.5% ± 0.0 and 99.8% ± 0.3 at all positions, 99.3% ± 1.3, 99.3 ± 1.3 and 100.0% ± 0.0 at DRM positions and 100.0% ± 0.0, 100.0% ± 0.0 and 100.0% ± 0.0 at SDRM positions. Drug resistance interpretations were concordant between all the replicates and the non-diluted plasma sample.

### Applicability of the test in routine clinical practice

To assess the amplification success rate, results obtained during routine clinical practice for 380 clinical samples from 352 different patients were evaluated. At first, the amplification was successful for 84% of the samples (321/380). For the 59 samples that could not be amplified in the first attempt, a second RNA aliquot from the same extraction was again subjected to the in-house amplification procedure and with this strategy another 19 samples could be amplified. Subsequently, for twenty of the samples that failed again in the amplification procedure and for which enough plasma was still available, a second extraction and amplification round was attempted. An additional 5 samples could be amplified, resulting into an overall amplification success rate of 91% (345/380).

Sequencing results covering 1,302 nucleotides (amino acids 1 in PR to 335 in RT), could be obtained for all amplified reactions (345/345). However, 3 sequences were rejected as quality control analysis suggested APOBEC editing, resulting in an overall sequencing success rate of 99% (342/345).

Phylogenetic analysis revealed that the subtype distribution among the 345 obtained sequences were 34.9% subtype B, 12.8% CRF19_cpx, 10.2% CRF20_BG, 9.0% CRF18_cpx, 9.0% URF, 7.8% subtype C, 7.0% CRF24_BG, 3.2% subtype G, 3.2% CRF23_BG, 0.9% subtype H, 0.6% subtype A, 0.6% subtype F, 0.6% CRF02_AG, 0.3% subtype J and 0.3% CRF31_BC.

An estimate of the cost of reagents and disposables was made. The overall cost per sample using this procedure was around € 80 per single genotyping attempt and increased to € 100 per successful genotype in our local testing scheme after initial amplification failure, which is still only half as expensive as the ViroSeq system. The working time cost and necessary equipment, such as pipettes, centrifuges, thermal cyclers, electrophoresis unit and automatic sequencer were excluded from these calculations.

### Sample mix-up or cross-contamination

The negative control added to each run was negative, suggesting the absence of cross-contamination and sample mix-up. In addition, none of the generated sequences revealed an unusual high number of mixed bases. Finally, a maximum likelihood tree was inferred using the sequences that passed the quality control assessment (S1 Fig.). Sequences generated from the same sample or from the same patient, consistently clustered together with a high bootstrap support, indicating the absence of sample mix-up or cross-contamination during sample processing.

## Discussion

The objective of this study was to design and validate an in-house system that could be implemented in Cuba, a resource limited setting with an HIV-1 epidemic with various subtypes and CRFs. To validate the here designed in-house system, the performance characteristics of the test were evaluated, including the determination of the lower limit of amplification and the assessment of accuracy (trueness and precision). Finally, the applicability of the test was evaluated in a clinical setting. Samples for validation experiments were carefully selected, so that the overall subtype distribution of the panels reflected the diversity in the Cuban epidemic. In addition, samples displaying a broad range of viral loads and complex drug resistance profiles were chosen when possible.

For HIV-1 genotypic drug resistance testing, no gold standard is available. Therefore, trueness was assessed by comparing the in-house assay with the ViroSeq Genotyping System version 2.0. The use of this commercial test as a reference standard has been frequently described in previous studies [12,22–32] and is recommended in the WHO guidelines [9]. Although ViroSeq has been approved for HIV-1 subtype B strains only, and difficulties with non-B subtypes have been published earlier [33–35], other studies report that ViroSeq is also applicable to various HIV-1 subtypes and recombinant strains [29,36–39].

The acceptance criteria defined in advance were met for all the evaluated performance characteristics, demonstrating a high degree of accuracy of the in-house assay. The in-house assay was able to amplify samples with low-level viremia, ranging from 84 to 529 RNA copies/mL. Although only a limited number of samples have been included in this analysis, it suggests that the lower limit of amplification is well below the threshold of 1,000 RNA copies/mL for the diverse strains circulating in Cuba. The in-house assay showed very good agreement with ViroSeq on NT level (98.7% ± 1.0) and minor differences were mainly caused by base mixtures. ViroSeq detected more base mixtures than the in-house assay, suggesting an increased sensitivity of ViroSeq in detecting NT mixtures, which has previously been reported for another in-house method by Saravanan et al. [31]. Little AA differences between the two methods were observed at SDRM (3 of 602 or 0.5%) and DRM (13 of 1,244 or 1.0%) positions and were all caused by mixtures. The number of viral mixtures detected by the in-house method (6/13 at DRM and 2/3 at SDRM positions) was comparable with the number of mixtures detected by ViroSeq (7/13 at DRM and 1/3 at SDRM positions). Other published in-house assays show the tendency of detecting less mixtures then the ViroSeq assay [27,28]. In only 2 of the 14 samples, the drug resistance patterns for RT inhibitors slightly changed due to 2 DRM (RT 67N and RT 221Y) detected by ViroSeq, but not by the in-house method. For these samples, the viral load was close to the recommended amplification limit (1,413 and 1,660 copies/mL) of 1,000 RNA copies/mL. The ad hoc reviewing of electropherograms revealed that both discordances could be attributed to differences in editing strategies, as mutations were represented by a very small secondary peak (<10%) [40]. In this analysis, trueness was assessed by comparing the results of the in-house assay with only one sequence determination by ViroSeq. Therefore, it would be recommendable that the IPK could participate in an external quality assessment program in the future. In these programs, the true value is a consensus sequence that is derived from the results of different labs contributing to the program, which increases the certainty about the presence of a DRM. For this study, discordances between drug resistance patterns from ViroSeq and in-house were negligible, demonstrating the ability of the in-house method to detect clinically relevant mutations correctly. As a result, the in-house assay performed well in comparison with the reference standard ViroSeq.

A disadvantage of the in-house assay is that the risk for cross-contamination is higher than with ViroSeq, as it uses a nested PCR and no dUTP/UNG contamination control. This required more attention to avoid cross-contamination and the introduction of stringent precautions and quality control, such as the inclusion of a negative control in every run. The negative results of the negative controls in each run, the good quality of the sequences, the high specificity of the in-house assay as here assessed, and the phylogenetic results indicate that cross-contamination or sample mix-up had not happened using the implemented laboratory procedures.

Furthermore, the intra- (repeatability) and inter-assay variability (reproducibility) were evaluated by generating different replicates per sample in the same run by the same technician and in different runs by different technicians respectively, both under similar assay conditions. Minimal differences were observed between resistance patterns of replicates of the same sample and NT sequence identities were excellent, even at viral loads below 1,000 RNA copies/mL. The good results for precision can be explained by the strict adherence to good laboratory practices and is an indication for the robustness of the assay.

Subsequently, the in-house assay was evaluated in a clinical setting. Although the test was rather successful in routine practice and displayed a very good sensitivity for a broad range of HIV-1 subtypes, there is still a need for improvement as 16% of the samples could not be amplified in a single attempt. Although general guidelines for the pre-analytical steps and detailed standard operating procedures for the analytical steps do exist, deviations from these rules at some instances could not be excluded and could have contributed to the amplification failures. Furthermore, it is possible that low viral loads impacted the amplification success rate negatively. However, viral load tests are not systematically performed for all patients in Cuba, and if done, mostly not on the same isolation date as for genotyping, so viral load data were not available. Additionally, primers were designed in a way that they bind to the diverse strains, but strain specific mismatches at the primer binding sites could not be investigated for the amplification failures as these patients had not been tested yet with alternative methodologies and no sequences were yet available. Therefore, in future studies performance should be verified on a broad spectrum of samples with known viral load and quality indicators should be monitored regularly.

Of the samples for which an amplicon could be generated, 99% were sequenced successfully using the in-house method. Generated sequences covered codons 1–99 of PR and 1–335 of RT, which is more than the minimal region for which sequence information must be collected according to the WHO guidelines [9].

The in-house test provided a more affordable alternative to commercial assays as cost for reagents and disposables was less than half that of the ViroSeq HIV-1 Genotyping System [31]. Expensive equipment not yet available at the IPK and necessary for HIV-1 genotyping, was purchased at the end of 2008 with the financial support of VLIR and Global Fund To Fight AIDS, Tuberculosis and Malaria. As these instruments were versatile, the investments did not only enable the implementation of HIV-1 drug resistance testing in Cuba but also contributed to the capacity building of the entire microbiology department. Although the financial burden of drug resistance testing is still substantially, the lower consumables costs of the in-house procedure enabled the implementation of drug resistance testing in this resource-limited setting. After training of the personnel, the in-house assay was implemented gradually in 2009, initially using retrospective samples to corroborate the good functioning of the system. Later in 2009, the assay was introduced in routine clinical practice for HIV-1 patients attending the IPK and for whom a resistance test was required.

In conclusion, this study describes the design, evaluation and implementation of an accurate and affordable HIV-1 drug resistance assay that is applicable to various subtypes and CRFs in Cuba. The assay made the assessment of drug resistance against PR inhibitors and RT inhibitors possible for individual patient management as well as in surveillance studies. This is an important weapon against the accumulation of drug resistance in HIV-1 patients failing antiviral therapy and the subsequent spread to newly infected patients.

## Supporting Information

S1 Fig Midpoint rooted maximum likelihood tree (1,000 bootstraps) of the sequences generated for the performance evaluation of the in-house assay. Trueness-IH and-VS sequences generated with respectively the in-house and ViroSeq assay to evaluate trueness (green); PC Positive Control; Reprod-A, -B and -C Replicates generated to evaluate reproducibility (red); Repeat-A, -B and -C Replicates generated to evaluate repeatability (blue); The subtypes and CRF reference dataset (black). Branches whithout taxa represent sequences for the evaluation of applicability. Only bootstrap values >70 are shown.
(PDF)
