# Supplementary material for: Performance of an In-House Human Immunodeficiency Virus Type 1 Genotyping System for Assessment of Drug Resistance in Cuba
Source: PLoS One. 2015 Feb 11;10(2):e0117176. doi: 10.1371/journal.pone.0117176 (PMC4324769; doi:10.1371/journal.pone.0117176)
Supplement: S1 Fig — Trueness-IH and-VS sequences generated with respectively the in-house and ViroSeq assay to evaluate trueness (green); PC Positive Control; Reprod-A, -B and -C Replicates generated to evaluate reproducibility (red); Repeat-A, -B and -C Replicates generated to evaluate repeatability (blue); The subtypes and CRF reference dataset (black). Branches whithout taxa represent sequences for the evaluation of applicability. Only bootstrap values >70 are shown. (PDF) [file pone.0117176.s001.pdf]

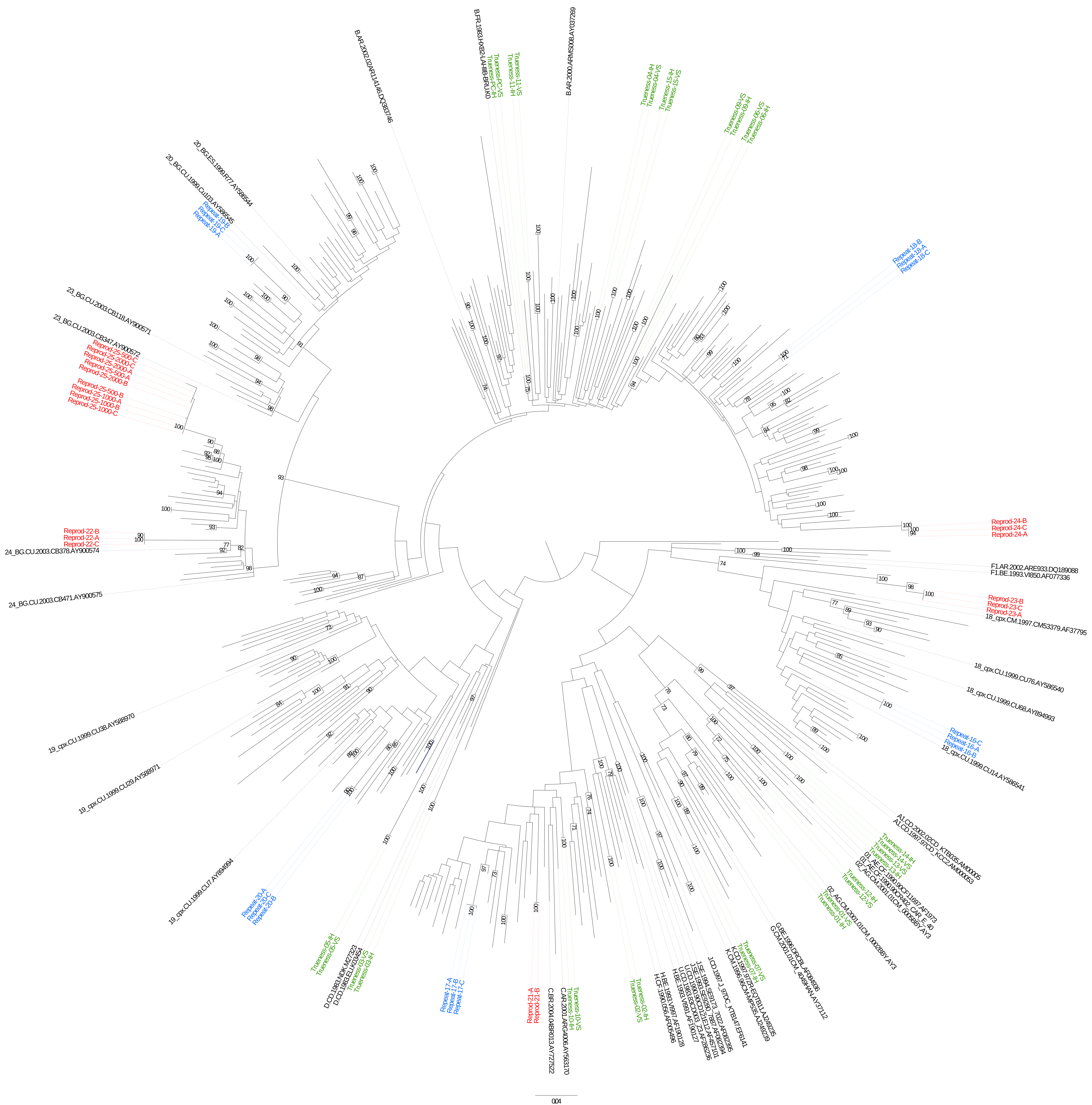

B.AR.2002.02AR114146.DQ383746
B.FR.1983.HXB2-LAI-IIIB-BRU.K0
Trueness-PC-VS
Trueness-PC-IH
Trueness-11-IH
Trueness-11-VS
B.AR.2000.ARMS008.AY037269
Trueness-04-IH
Trueness-04-VS
Trueness-15-IH
Trueness-15-VS
Trueness-09-VS
Trueness-09-IH
Trueness-06-VS
Trueness-06-IH
Repeat-18-B
Repeat-18-A
Repeat-18-C
Reprod-24-B
Reprod-24-C
Reprod-24-A
F1.AR.2002.ARE933.DQ189088
F1.BE.1993.VI850.AF077336
Reprod-23-B
Reprod-23-C
Reprod-23-A
18_cpx.CM.1997.CM53379.AF37795
18_cpx.CU.1999.CU76.AY586540
18_cpx.CU.1999.CU68.AY894993
Repeat-16-C
Repeat-16-A
Repeat-16-B
18_cpx.CU.1999.CU14.AY586541
A1.CD.2002.02CD_KTB035.AM000005
A1.CD.1997.97CD_KCC2.AM000053
Trueness-14-IH
Trueness-14-VS
Trueness-13-VS
Trueness-13-IH
01_AE.CF.1990.90CF11697.AF1973
01_AE.CF.1990.90CF402_CAR_E_40
02_AG.CM.2001.01CM_0005BBY.AY3
Trueness-12-IH
Trueness-12-VS
02_AG.CM.2001.01CM_0002BBY.AY3
Trueness-01-VS
Trueness-01-IH
G.BE.1996.DRCBL.AF084936
G.CM.2001.01CM_4049HAN.AY37112
K.CD.1997.97ZR_EQTB11.AJ249235
K.CM.1996.96CM_MP535.AJ249239
Trueness-07-VS
Trueness-07-IH
J.CD.1997.J_97DC_KTB147.EF6141
J.SE.1994.SE9173_7022.AF082394
J.SE.1993.SE9280_7887.AF082395
U.CD.1983.83CD003_Z3.AF286236
U.CD.1990.90CD121E12.AF457101
H.BE.1993.VI991.AF190127
H.BE.1993.VI997.AF190128
H.CF.1990.056.AF005496
Trueness-02-IH
Trueness-02-VS
Trueness-10-VS
Trueness-10-IH
C.AR.2001.ARG4006.AY563170
C.BR.2004.04BR013.AY727522
Reprod-21-A
Reprod-21-B
Repeat-17-A
Repeat-17-B
Repeat-17-C
Trueness-05-IH
Trueness-05-VS
D.CD.1983.NDK.M27323
D.CD.1983.ELI.K03454
Trueness-03-VS
Trueness-03-IH
Repeat-20-A
Repeat-20-C
Repeat-20-B
19_cpx.CU.1999.CU7.AY894994
19_cpx.CU.1999.CU29.AY588971
19_cpx.CU.1999.CU38.AY588970
24_BG.CU.2003.CB471.AY900575
24_BG.CU.2003.CB378.AY900574
Reprod-22-B
Reprod-22-A
Reprod-22-C
Reprod-25-500-B
Reprod-25-1000-A
Reprod-25-1000-B
Reprod-25-1000-C
Reprod-25-500-C
Reprod-25-2000-C
Reprod-25-2000-A
Reprod-25-500-A
Reprod-25-2000-B
23_BG.CU.2003.CB347.AY900572
23_BG.CU.2003.CB118.AY900571
Repeat-19-B
Repeat-19-C
Repeat-19-A
20_BG.CU.1999.CU103.AY586545
20_BG.ES.1999.R77.AY586544
0.04
